# Hormone replacement therapy alone or in combination with tamoxifen in women with thin endometrium undergoing frozen-thawed embryo transfer: A retrospective study

**DOI:** 10.3389/fendo.2023.1102706

**Published:** 2023-03-01

**Authors:** Qingqing Shi, Chenyang Huang, Jingyu Liu, Yifan Li, Na Kong, Jie Mei, Xiaoyue Shen, Yanxin Sun, Feifei Lu, Haixiang Sun, Guijun Yan

**Affiliations:** ^1^ Center for Reproductive Medicine and Obstetrics and Gynecology, Drum Tower Clinic Medical College of Nanjing Medical University, Nanjing, China; ^2^ Center for Reproductive Medicine and Obstetrics and Gynecology, Nanjing Drum Tower Hospital, Nanjing University Medical School, Nanjing, China; ^3^ Center for Molecular Reproductive Medicine, Nanjing University, Nanjing, China

**Keywords:** thin endometrium, tamoxifen, hormone replacement, pregnancy outcomes, frozen-thawed embryo transfer

## Abstract

**Research question:**

To investigate the effects of two protocols (hormone replacement therapy (HRT) alone or in combination with tamoxifen) on the endometrium and pregnancy outcome of patients with thin endometrium in frozen-thawed embryo transfer (FET) cycles.

**Design:**

A total of 465 infertile patients with thin endometrium who underwent FET between January 2020 to June 2021 at the Drum Tower Hospital affiliated with Nanjing University Medical School were retrospectively analyzed. A total of 187 patients were given tamoxifen in addition to HRT (TMXF-HRT group), whereas 278 patients were given only HRT (HRT group). Clinical data were compared between the two groups, including general characteristics, endometrial thickness, and clinical pregnancy outcomes.

**Results:**

There were no significant differences in baseline characteristics of all enrolled patients between two groups. Serum progesterone (P) was higher in HRT group than in the TMXF-HRT group (0.28 ± 0.53 ng/mL *vs*. 0.15 ± 0.25 ng/mL, P = 0.002). There was a significant increase in endometrial thickness in the TMXF-HRT group compared with the HRT group (OR: 1.54, 95% CI: 1.32-1.75, P < 0.001). There were no significant differences in the clinical pregnancy rate, embryo implantation rate, early miscarriage rate, or live birth rate between these two groups.

**Conclusion:**

Although tamoxifen when used in combination with hormone replacement therapy can significantly increase endometrial thickness, it may not have a role in improving the pregnancy outcomes of patients with thin endometrium undergoing FET cycles.

## Introduction

The first patient who underwent frozen-thawed embryo transfer (FET) had a successful pregnancy in 1983. Recently, with the continuous improvement of vitrification solutions, FET has become a routine embryo transfer strategy for assisted reproductive technology (ART). Frozen-thawed embryo transfer plays important roles in preimplantation genetic testing (PGT), elevated progesterone levels, prevention of ovarian hyperstimulation syndrome (OHSS), selective single embryo transfer, decreased ovarian reserve, and in adenomyosis patients (1). The pregnancy outcome of a FET cycle is affected by many factors, such as female age, number and quality oftransferred embryos, endometrial receptivity, and maternal conditions. Endometrial receptivity is widely regarded as a key factor for successful FET cycles. There are several evaluation indicators of endometrial receptivity including molecular biology, morphology, and proteomics markers. Endometrial thickness, an evaluation indicator of morphology, is very important for embryo implantation. Endometrial thickness can be easily measured by ultrasound examination and has become a commonly used clinical evaluation index for endometrial receptivity (2–4).

According to previous research, the incidence of thin endometrium in ART cycles is between 1.5% and 9.1% (5, 6), even though several treatments have been attempted to preserve endometrial thickness. Treatments consisting of the administration of exogenous estrogen, vitamin E, aspirin, intravaginal sildenafil citrate and pentoxifylline, stem cells and growth hormone have been employed to improve the endometrial thickness; however, these treatments are still controversial, and their therapeutic effects have been satisfactory. A thin endometrium is still a difficult problem to manage in embryo transfer cycles (7–10), and an effective solution still needs to be discovered.

Studies have confirmed that tamoxifen can upregulate the expression of estrogen receptors to promote the activation of estrogen-related gene pathways and stimulate the growth of endometrial cells (11–13). Tamoxifen can induce the expression of endometrial vascular endothelial growth factor (VEGF) in the thin endometrium and promote the formation of endometrial microvessels. In addition, tamoxifen can also promote an increase in endometrial stromal cell paracrine factors, upregulate insulin-like growth factors, and stimulate endometrial epithelial proliferation (14, 15). Tamoxifen has been frequently employed in the ovulation induction cycle, but rarely in the hormone replacement therapy (HRT) cycle. Considering the upregulation of estrogen receptors by tamoxifen, we sought to administer tamoxifen in combination with HRT in the FET cycle, but its clinical efficacy is not clear at present.

The aim of our study was to determine the efficacy of using tamoxifen in combination with HRT on thin endometrium to select an optimal endometrial preparation protocol for patients with thin endometrium undergoing FET cycles, which could improve endometrial thickness and pregnancy outcomes.

## Materials and methods

### Study population

Data from patients who underwent the suggested FET cycle at the Reproductive Medicine Centre, Nanjing Drum Tower Hospital affiliated with Nanjing University Medical School between January 2020 and June 2021 were selected for analysis. This study was approved by the institutional ethics committee (ethical approval number: 2019-224).

The inclusion criteria were as follows (1): maternal age less than 38 years, and basal serum follicle stimulating hormone (FSH) level less than 10 IU/L; (2) endometrial thickness less than 8 mm in at least two FET cycles with either endometrial preparation (natural cycle, HRT cycle, ovarian stimulation cycle); and (3) at least one high-quality embryo for transfer. The quality of cleavage-stage embryos was evaluated from three aspects: cell number, fragmentation, and symmetry. High-quality cleavage-stage embryos had 8–10 cells, a fragment proportion of less than 5%, and symmetrical blastomeres. Blastocysts were rated using the Gardner grading system. High-quality blastocysts had an expansion grade higher than III, and inner cell mass (ICM) and trophectoderm (TE) score of at least grade B (16).

Exclusion criteria: (1) abnormal chromosome karyotype; (2) presence of other uterine diseases, such as a uterine myoma that affects the uterine cavity, adenomyosis, endometriosis, congenital uterine malformations, endometrial tuberculosis, etc.; (3) contraindications to HRT; (4) participation in other clinical studies; and (5) a history of previous fundus diseases.

### Procedure

**Figure 1:**
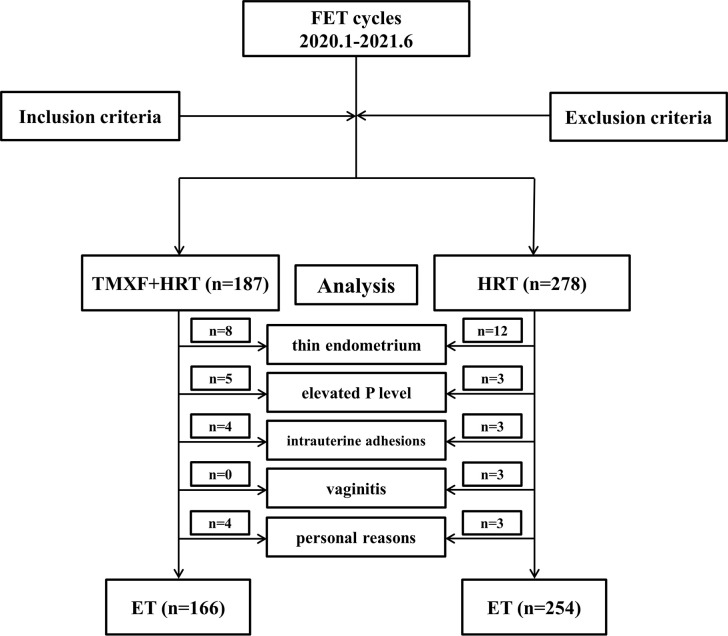
Flow chart of the patient inclusion and exclusion criteria.

A total of 465 patients who met the inclusion criteria were divided into two groups. Patients receiving tamoxifen in addition to HRT were defined as the TMXF-HRT group (n = 187), 166 of whom had embryos transferred. Patients receiving HRT alone were defined as the HRT group (n = 278), 254 of whom had embryos transferred. A total of 45 patients gave up the planned FET cycle for many reasons: 20 patients (8 in TMXF-HET group and 12 in HRT-group) refused to transfer because of the thin endometrium; 8 patients (5 in TMXF-HET group and 3 in HRT-group) had elevated serum progesterone levels for unknown reasons; vaginal ultrasound of 7 patients (4 in TMXF-HET group and 3 in HRT-group) showed intrauterine adhesions; 3 patients in HRT group suffered from vaginitis; 7 patients (4 in TMXF-HET group and 3 in HRT-group) refused to transfer due to personal reasons. A flow chart depicting this study is shown in **Figure 1**.

The patients took Femoston (estradiol 2 mg po ter in die (t.i.d.)) from the day 2 of the menstrual cycle. In addition, patients in the TMXF-HRT group received tamoxifen (10 mg po bis in die (b.i.d.)) for 5 days from day 2 of the menstrual cycle. Patients in both groups underwent endometrial thickness and serum hormone (estrogen and progesterone) measurements on day 10. Two other procedures were used listed for further treatment depending on endometrial thickness:

(1) If endometrial thickness was more than 8 mm, patients continued to take Femoston (estradiol 2 mg po t.i.d.). On the day 19 of the menstrual cycle, progesterone was given to the women to transform the endometrium to the secretory phase.

(2) If endometrial thickness was less than 8 mm, oral Femoston (estradiol tablet) was administered at 8 mg per day. On day 19 of the menstrual cycle, progesterone was given to transform the endometrium to the secretory phase. Endometrial transformation and corpus luteum support: On day 18 of the menstrual cycle, patients were given oral Femoston (1 mg estradiol plus 10 mg dydrogesterone tablet, po t.i.d.) and intramuscular progesterone (60 mg quaque die (q.d.)). Cleavage-stage embryos were transferred on the day 5 and blastocysts were transferred on the day 6 after endometrial transformation. An ultrasound examination was performed 30 days after embryo transfer. Luteal support was continued until 55-60 days after embryo transfer.

Patient data, including age, endometrial thickness, E_2_ level and P level before progesterone administration were collected, and the change in endometrial thickness was defined as endometrial thickness changes in the same patient during the current and previous FET cycles. Serum β-human chorionic gonadotropin (β- hCG) levels were measured 2 weeks after embryo transfer to determine biochemical pregnancy. Patients with elevated serum β-hCG levels were examined by transvaginal ultrasound 4 weeks after embryo transfer to confirm the clinical pregnancy and number of implanted embryos. Clinical pregnancy is defined as the presence of a gestational sac. Early miscarriage was defined as a miscarriage occurring within 12 weeks of gestation. Live birth was defined as the delivery of a healthy infant after 28 weeks of gestation.

### Statistical analysis

The general data are presented as the mean ± SD and n (%). For comparisons between two groups, the independent sample Kruskal-Walli’s test was used for continuous variables and the Mantel-Haenszel test for trend was used for categorical variables. Multiple regression analysis was used to analyze the association between tamoxifen and endometrial thickness. We constructed two adjusted models: (1) adjusted for female age; and (2) adjusted for female age, E_2_ levels, and P levels before endometrial transformation. A two-sided P < 0.05 was considered statistically significant, and confidence intervals (CIs) were set at 95%. All analyses were performed with the statistical software SPSS 23.0, Prism 7.0, package R (http://www.R-project.org, The R Foundation) and EmpowerStats (www.empowerstats.com, X & Y solution, Inc. Boston, MA).

## Results

### Baseline characteristics of patients enrolled in this study

Baseline information of patients in the HRT and TMXF-HRT groups is shown in **Table 1**. In total, 278 and 187 patients were enrolled in the HRT group and TMXF-HRT group, respectively. There were no significant differences in age, baseline follicle-stimulating hormone (FSH), antral follicle count (AFC), anti-mullerian hormone (AMH), infertility duration, infertility type, body mass index (BMI) and E_2_ level before endometrial transformation between the two groups. Serum P level before endometrial transformation in the HRT group was higher than those in the TMXF-HRT group (0.28 ± 0.53 ng/mL *vs.* 0.15 ± 0.25 ng/mL, *P* = 0.002). Patients in the TMXF-HRT group (7.99 ± 1.32 mm) possessed a thicker endometrium than those in the HRT group (7.00 ± 0.61 mm, *P* < 0.001). There was a significant increase in endometrial thickness in the TMXF-HRT group (1.23 ± 1.16 mm *vs.* -0.33 ± 1.07 mm, *P* < 0.001).

**Table 1:** General data in HRT cycles versus tamoxifen in combination with HRT cycles.

	HRT	TMXF-HRT	*P* value
Number (n)	278	187	
Female age (years)	33.6 ± 4.6	32.9 ± 4.2	0.089
BMI	22.78 ± 3.00	22.64 ± 3.06	0.622
FSH	7.90 ± 2.90	7.58 ± 3.20	0.264
AMH	3.35 ± 4.51	3.01 ± 2.48	0.344
AFC	15.7 ± 7.8	16.6 ± 7.5	0.249
Infertility duration (years)	3.4 ± 2.6	3.1 ± 2.0	0.193
Infertility type (primary/secondary)	90/188	56/131	0.583
E_2_ (pg/mL)	1268.62 ± 1165.03	1225.94 ± 965.65	0.679
P (ng/mL)	0.28 ± 0.53	0.15 ± 0.25	0.002
Em (mm)	7.00 ± 0.61	7.99 ± 1.32	<0.001
Different Em groups			N/A
≤ 6 mm	35 (12.59%)	12 (6.42%)	
> 6 mm, ≤ 7 mm	114 (41.00%)	41 (21.92%)	
> 7 mm, ≤ 8 mm	120 (43.17%)	53 (28.34%)	
> 8 mm	9 (3.24%)	81 (43.32%)	
Change in Em (mm)	-0.33 ± 1.07	1.23 ± 1.16	<0.001

FSH, follicle-stimulating hormone; AFC, antral follicle count; AMH, anti-mullerian hormone; BMI, body mass index; E_2_, estrogen (before endometrial transformation); P, progesterone (before endometrial transformation); Em, endometrial thickness.

### Association between endometrial thickness and tamoxifen supplementation

In **Table 2**, an adjusted analysis of the association between endometrial thickness and tamoxifen supplementation showed that tamoxifen supplementation resulted in a 3% reduction in endometrial thickness after adjusting for female age (OR: 0.97, 95% CI: 0.79-1.14, *P* < 0.001). After adjusting for E_2_ and P levels, there was no significant change in endometrial thickness as a result of tamoxifen supplementation (OR: 1.00, 95% CI: 0.82-1.18, *P* < 0.001). To determine the impact of tamoxifen on endometrial thickness more accurately, we further compared the changes in endometrial thickness for TMXF-HRT patients between their TMXF-HRT cycle and previous FET cycle. As shown in **Table 3**, an adjusted analysis of the association between endometrial thickness change and tamoxifen supplementation indicated that tamoxifen supplementation resulted in a 54% increase in endometrial thickness compared to HRT alone for any given patient (OR: 1.54, 95% CI: 1.32-1.75, *P* < 0.001).

**Table 2:** Multivariate analysis for the effect of tamoxifen supplementation on the endometrial thickness between the TMXF-HRT group and control group.

Variable	Adjustment I			Adjustment II		
	Odds ratio	95% CI	*P* value	Odds ratio	95% CI	*P* value
tamoxifen supplementation	0.97	0.79, 1.14	<0.001	1.00	0.82, 1.18	<0.001

Adjustment I for, female age; Adjustment II for, E_2_ and P levels before endometrial transformation.

**Table 3:** Multivariate analysis for the effect of tamoxifen supplementation on the change in endometrial thickness between TMXF-HRT cycles and last FET cycles.

Variable	Adjustment I			Adjustment II		
	Odds ratio	95% CI	*P* value	Odds ratio	95% CI	*P* value
tamoxifen supplementation	1.56	1.35, 1.77	<0.001	1.54	1.32, 1.75	<0.001

Adjustment I for, female age; Adjustment II for, E_2_ and P levels before endometrial transformation.

### Tamoxifen supplementation on clinical pregnancy outcomes

We collected baseline information and clinical pregnancy outcomes for patients who underwent FET in this study. In total, 254 out of 278 patients in the HRT group underwent FET cycles, while the rate in the TMXF-HRT group was 166/187 (**Table 4**). Overall, there was no difference in female age and the number or type of transferred embryos between the HRT-FET group and the TMXF-HRT-FET group. Patients in the HRT-FET group had higher E_2_ (1479.30 ± 1137.14 pg/mL *vs.* 1240.69 ± 962.27 pg/mL, *P* = 0.026) and P (0.23 ± 0.24 ng/mL *vs.* 0.13 ± 0.11 ng/mL, *P* < 0.001) levels than those in the TMXF-HRT-FET group. In addition, the endometrial thickness of patients in the TMXF-HRT-FET group was greater than that of patients in the HRT-FET group (8.13 ± 1.29 mm *vs.* 7.21 ± 0.50 mm, *P* < 0.001). There were no significant differences in the clinical pregnancy rate, early miscarriage rate, or live birth rate between these two groups. The embryo implantation rate was higher in the HRT-FET group (37.75% *vs.* 27.97%, *P* = 0.014). An adjusted analysis of the association between tamoxifen supplementation and clinical pregnancy outcomes showed no significant difference in embryo implantation rate and clinical pregnancy rate after tamoxifen supplementation (**Table 5**).

**Table 4:** General data for HRT-FET cycles versus TMXF-HRT-FET cycles.

	HRT-FET	tamoxifen-HRT-FET	*P* value
Number (n)	254	166	
Female age (years)	32.5 ± 4.5	32.7 ± 4.2	0.704
E_2_ (pg/mL)	1479.30 ± 1137.14	1240.69 ± 962.27	0.026
P (ng/mL)	0.23 ± 0.24	0.13 ± 0.11	<0.001
Em (mm)	7.21 ± 0.50	8.13 ± 1.29	<0.001
No. of transferred embryos (n)	1.37 ± 0.48	1.42 ± 0.50	0.255
Type of transferred embryo (cleavage-stage embryo/blastocyst)	125/129	82/84	0.973
clinical pregnancy rate (%)	42.52 (108/254)	34.34 (57/166)	0.093
embryo implantation rate (%)	37.75 (131/347)	27.97 (66/236)	0.014
Early miscarriage rate (%)	19.44 (21/108)	15.79 (9/57)	0.563
live birth rate (%)	31.10 (79/254)	24.10 (40/166)	0.119

E_2_, estrogen (before endometrial transformation); P, progesterone (before endometrial transformation); Em, endometrial thickness.

**Table 5:** Multivariate analysis for the effect of tamoxifen supplementation on the clinical outcome of total FET cycles.

Dependent Variable	Adjustment I			Adjustment II		
	β/OR	95% CI	*P* value	β/OR	95% CI	*P* value
Embryo Implantation Rate	-0.09	-0.18, -0.01	0.033	-0.09	-0.18, -0.00	0.056
Clinical Pregnancy Rate	0.71	0.47, 1.07	0.104	0.72	0.47, 1.09	0.123

Adjustment I for, female age; Adjustment II for, E_2_ and P levels before endometrial transformation.

## Discussion

Our study innovatively explored the improvement effect of tamoxifen in combination with HRT on a thin endometrium. The results suggest that administering tamoxifen with HRT can significantly increase the endometrial thickness of patients. Therefore, tamoxifen plus HRT is an optional endometrial preparation protocol in FET cycles for patients with thin endometrium. However, the study results also suggest that this protocol has no significant improvement effect on the clinical pregnancy outcome, so further prospective research is needed to explore the precise clinical efficacy of the TMXF-HRT protocol in FET cycles for patients with thin endometrium.

Embryo implantation requires a cross talk between the embryo and the receptive endometrium. Especially during frozen-thawed embryo transfer cycles, endometrial receptivity is widely considered to be a key factor for ART outcomes. The definition of thin endometrium varies and is mostly described as an endometrial thickness < 7 mm or < 8 mm in the mid-luteal phase, the etiologies of which include the uterine surgery, uterine tuberculosis, endocrine factors, congenital abnormal uterine development, in addition to some unknown causes. Some previous studies have found that the clinical pregnancy rate and live birth rate of patients with thin endometrium are significantly reduced, while other studies have shown that the live birth rate is not significantly decreased (17, 18). In 2018, a study of more than 40,000 cycles suggested that the clinical pregnancy rate and live birth rate of patients with endometrial thickness less than 8 mm in fresh embryo transfer cycles or less than 7 mm in FET cycles were significantly reduced with the decrease in endometrial thickness (19). Although the cutoff values of endometrial thickness that could affect pregnancy rate were different in various studies, the influence of a thin endometrium on the pregnancy outcome was clear (7, 20, 21).

Endometrial preparation is a key step in FET cycles, the purpose of which is to promote endometrial growth with endogenous or exogenous estrogen and then transform the endometrium to the secretory phase by administering progesterone, so that it can successfully receive an embryo for implantation. Conventional endometrial preparation schemes for FET mainly include a natural cycle/modified natural cycle, hormone replacement cycle and ovarian stimulation cycle. A number of studies have proven that none of them has a clear advantage (22), and the most appropriate scheme is often selected according to the patient’s characteristics. Currently, the commonly used endometrium preparation protocols for patients with thin endometrium include hormone replacement therapy (HRT), human menopausal gonadotropin (HMG) and tamoxifen. Patients undergoing hormone replacement cycles have the flexibility of time, the advantages of good compliance, and a lower cycle cancellation rate. However, in addition to the common side effects of estrogen, such as nausea, breast tenderness and headaches, large doses of estrogen can induce liver damage, increase the tendency for blood clotting, and even lead to deep vein thrombosis. Tamoxifen can be used in ovulation induction cycles, and HMG can be added when necessary. Compared with the HRT cycle, the use of tamoxifen requires increased ultrasound monitoring and serum hormone examinations in the ovarian stimulation cycle and an increase in the number of patients’ medical visits. In addition, HMG administration is less comfortable and convenient than the administration of oral medication in the HRT cycle. Moreover, multifollicular development will lead to a higher risk of ovarian hyperstimulation syndrome (OHSS) which should be avoided in FET cycles.

Tamoxifen has been used in ART treatment for a long time. It is mainly used for ovulation induction in patients with ovulation disorders, thin endometrium, and breast diseases. The main mechanism of tamoxifen action is related to its ability to bind to estrogen receptors. Estrogen receptors have both genomic (nuclear initiated steroid signaling) and nongenomic (membrane-initiated steroid signaling) signaling pathways, which are activated when estrogen binds to its receptors. Therefore, various physiological processes are regulated (23, 24). Through their genomic activity, estrogen receptors act as ligand-dependent transcription factors and regulate the expression of multiple genes. Estrogen is the main stimulating factor for endometrial growth. In the HRT cycle of patients with thin endometrium, endometrial growth is not evident under a high dosage of estrogen, which may be related to a lack of estrogen receptor activity (25).

There are various molecular mechanisms by which tamoxifen promotes endometrial growth. While stimulating follicles to develop and produce estrogen, tamoxifen mainly shows estrogen-stimulating effects in the endometrium. Many studies have shown that tamoxifen for ovulation induction therapy results in better endometrial thickness (26, 27). The results indicated that tamoxifen had certain advantages for patients with thin endometrium. The previous application of tamoxifen in ART treatment focused on its function on ovarian stimulation. This study combined tamoxifen with conventional HRT to induce endometrial estrogen receptors before exogenous estrogen supplementation. Tamoxifen can increase the local activity of exogenous estrogen in the endometrium and stimulate the proliferation of endometrial cells and angiogenesis. At the same time, it can reduce the dosage of exogenous estrogen and increase endometrial thickness.

This is the first time that TMXF has been combined with hormone replacement therapy in the treatment of patients with a thin endometrium. However, this study has some limitations. The specific embryo score was not included as an adjustment variable for statistical analysis. In addition, due to the limitations of our data system, it was difficult for us to directly obtain the corresponding data for oocyte-pick-up cycles (such as the number of eggs retrieved, the number of available embryos, etc.) and data on the cause of thin endometria for patients undergoing FET cycles. Therefore, we cannot determine whether these variables would affect the conclusions of this study. The retrospective design is the main shortcoming of this study. To further clarify the effects of tamoxifen used in combination with HRT, higher quality and large-scale randomized controlled trials are needed.

## Conclusion

Although tamoxifen in combination with hormone replacement therapy can significantly increase endometrial thickness, it may not have a role in improving the pregnancy outcomes of patients with thin endometrium undergoing FET cycles.

## Data availability statement

The raw data supporting the conclusions of this article will be made available by the authors, without undue reservation.

## Ethics statement

This study has been approved by the ethics committees of Nanjing Drum Tower Hospital (ethical approval number: 2019-224).

## Author contributions

QS, CH, JL, HS, and GY contributed to conception and design of the study. HS, YL, and NK organized the database. CH, JM, and YS performed the statistical analysis. QS and XS wrote the first draft of the manuscript. CH and FL wrote sections of the manuscript. All authors contributed to manuscript revision, read, and approved the submitted version.
